# Synthesis and Structure of 5-Methyl-9-(trifluoromethyl)-12*H*-quino[3,4-*b*][1,4]benzothiazinium Chloride as Anticancer Agent

**DOI:** 10.3390/molecules29184337

**Published:** 2024-09-12

**Authors:** Andrzej Zieba, Violetta Kozik, Kinga Suwinska, Agata Kawulok, Tadeusz Pluta, Josef Jampilek, Andrzej Bak

**Affiliations:** 1 Department of Organic Chemistry, Faculty of Pharmaceutical Sciences in Sosnowiec, Medical University of Silesia, Jagiellonska 4, 41-200 Sosnowiec, Poland; 2 Institute of Chemistry, University of Silesia, Szkolna 9, 40-007 Katowice, Poland; agata.hadrys@us.edu.pl (A.K.); tadeusz.pluta@gmail.com (T.P.); josef.jampilek@gmail.com (J.J.); andrzej.bak@us.edu.pl (A.B.); 3 Faculty of Mathematics and Natural Sciences, Cardinal Stefan Wyszynski University, K. Woycickiego 1/3, 01-938 Warszawa, Poland; k.suwinska@uksw.edu.pl; 4 Department of Bone Marrow Transplantation and Oncohematology, Maria Sklodowska-Curie National Research Institute of Oncology, Gliwice Branch, ul. Wybrzeze Armii Krajowej 15, 44-101 Gliwice, Poland

**Keywords:** phenothiazine, azaphenothiazines, anticancer activity, antiproliferative activity, X-ray analysis

## Abstract

In this work, the synthesis, structural analysis and anticancer properties of 5-methyl-9-trifluoromethyl-12*H*-quino[3,4-*b*][1,4]benzothiazinium chloride (**3**) are described. Compound **3** was synthesized by reacting 1-methyl-4-butylthio-3-(benzoylthio)quinolinium chloride with 4-(trifluoromethyl)aniline, respectively. The structure of the resulting product was determined using ^1^H-NMR and ^13^C-NMR spectroscopy as well as HR-MS spectrometry. The spatial geometry of agent **3** and the arrangement of molecules in the crystal (unit cell) were also confirmed using X-ray diffraction. The tetracyclic quinobenzothiazinium system is fairly planar because the dihedral angle between the planes formed by the benzene ring and the quinoline system is 173.47°. In order to obtain insight into the electronic charge distribution of the investigated molecule, electronic structure calculations employing the Density Functional Theory (DFT) were performed. Moreover, antiproliferative activity against a set of pancreatic cancer cell lines was tested, with compound **3** showing IC_50_ values against human primary pancreatic adenocarcinoma BxPC-3 and human epithelioid pancreatic carcinoma Panc-1 of 0.051 µM and 0.066 µM, respectively. The IC_50_ value of cytotoxicity/cell viability of the investigated compound assessed on normal human lung fibroblasts WI38 was 0.36 µM.

## 1. Introduction

Phenothiazine is a valid structural system commonly used in medicinal chemistry due to the revealed spectrum of biological properties [1,2,3,4,5,6]. As a matter of fact, phenothiazine derivatives are an excellent example of the impact that tiny structural changes can exert on the biological response. The analogues of phenothiazine were the first registered effective neuroleptic drug molecules [7]. The occurrence of an aminoalkyl subgroup in a thiazine nitrogen atom, especially when the interval between the thiazine nitrogen atom and the nitrogen atom of the substituent was three carbons, conditioned such biological potency. Interestingly, the reduction in N-thiazine and *N*-substituent distance to two carbon atoms resulted in an observed change in activity from neuroleptic to antihistamine, respectively.

**Figure 1:**
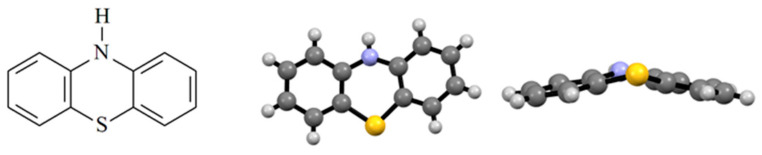
Structural formula and X-ray structure of 10*H*-phenothiazine.

In this context, the basic phenothiazine structural fragment has become a valuable scaffold for the synthesis of a number of phenothiazine derivatives exhibiting a variety of biological properties. Consequently, many phenothiazine derivatives have been described so far, revealing appealing biological properties, e.g., anticancer, antibacterial, anti-inflammatory, antiviral, etc. [8,9,10,11,12,13,14,15,16]. Not surprisingly, phenothiazine derivatives are also used within the drug repurposing strategy [17,18]. In practice, structural modifications of phenothiazine require the introduction of diverse pharmacophore groups in the thiazine nitrogen atom or carbon atoms of benzene rings [19,20]. On the other hand, another modification route relies on the replacement of one or both benzene rings with nitrogen heterocyclic systems, leading to the corresponding azaphenothiazines [21,22,23]. Structurally, the phenothiazine molecule is bent along an axis specified by the nitrogen and sulfur atoms of the thiazine ring, as shown in Figure 1 [24].

**Figure 2:**
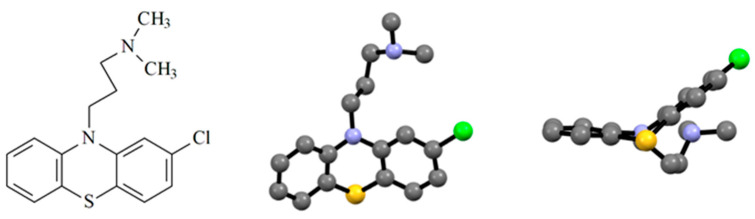
Structural formula and X-ray structure of chlorpromazine.

The dihedral angle between planes determined by the atoms of benzene rings is 145.92°, while the angle in the C-S-C thiazine ring is 100.86°, and the angle between the C-N-C bonds is 124.44°, respectively. The spatial structure of phenothiazine and azaphenothiazine derivatives depends on the nature of the substituents at the carbon atoms of the benzene rings or azine atoms and the thiazine nitrogen atom. As a matter of fact, chlorpromazine is known as the first effective phenothiazine neuroleptic containing an *N*,*N*-dimethylamine substituent at the thiazine nitrogen atom and a chlorine atom at position 2 of the phenothiazine system (see Figure 2) [25].

**Figure 3:**
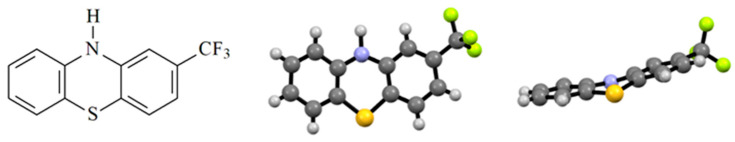
Structural formula and X-ray structure of 2-(trifluoromethyl)phenothiazine.

The tricyclic structure of chlorpromazine is noticeably much more folded compared to the phenothiazine molecule; the dihedral angle determined by the planes of the benzene rings reaches the value of 137.94°. In the case of a phenothiazine derivative substituted by a strongly electron-withdrawing trifluoromethyl (–CF_3_) group in position 2 of the ring, the tricyclic phenothiazine system is flatter, as illustrated in Figure 3. The dihedral angle specified by the planes of the benzene rings is 167.8° [26].

Details of the synthesis of the tetracyclic quinobenzothiazinium derivatives have been reported previously [27]. Briefly, the method involves the cyclization of betaine systems with the structure of 1-alkyl-4-(phenylamino)quinolinio-3-thiolates. The cyclization reaction occurs by replacing a hydrogen or halogen atom with a thiolate sulfur atom. Compounds of this type are generated in the reaction of thioquinanthrenium salts with aromatic amines or the reaction of 1-alkyl-4-alkylthio-3-acylthioquinolinium salts with aromatic amines [28,29].

It should be emphasized that the synthesized tetracyclic quinobenzothiazinium derivatives revealed noteworthy anticancer and antimicrobial properties as well [30,31]. Thus, further structural modifications of the tetracyclic quinobenzothiazine system are being investigated meticulously in order to unveil analogues with desired biological properties. The quinobenzothiazine scaffold was modified by introducing various types of pharmacophore groups at different positions of the tetracyclic quinobenzothiazine system. This created the possibility of a more effective interaction of the potential drug with the molecular target. Although very interesting correlations were observed, derivatives with an IC_50_ value lower than in the order of µM were not obtained [21,29,30,31,32]. The results obtained so far indicate that the most probable mechanism of anticancer activity of tetracyclic quinobenzothiazine compounds is DNA intercalation [21,29,30]. The presence of flat structural fragments in the molecule seems to be the preferable factor for this kind of action; therefore, we decided to modify the current direction of our research.

The principal aim of the proposed studies was to obtain derivatives containing a flat tetracyclic system, which would enable the formation of stable drug–DNA complexes, promoting stronger antiproliferative activity.

## 2. Results and Discussion

### 2.1. Chemistry–Design and Synthesis

**Scheme 1:**
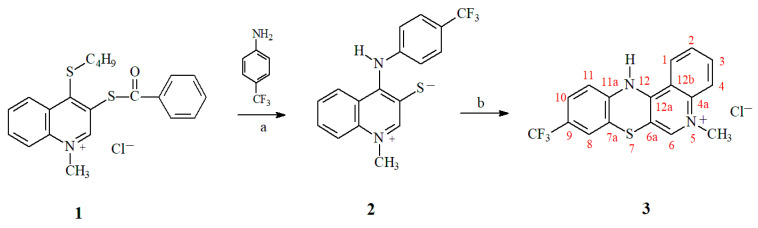
Reactions of salt **1** with 4-(trifluoromethyl)aniline. Reagents and conditions: (a) pyridine at 80 °C; (b) pyridine with HCl and air at 80 °C.

Structural characterization of new compounds (e.g., potential therapeutics) is a valid step of the contemporary search for potent anticancer agents [26,27]. Hence, the synthesis of the trifluoromethyl quinobenzothiazinium derivative (**3**) is presented with its structural analysis as well as its anticancer properties. The reaction of 1-methyl-4-butylthio-3-(benzoylthio)quinolinium chloride (**1**) with 4-(trifluoromethyl)- aniline gave 5-methyl-9-(trifluoromethyl)-12*H*-quino[3,4-*b*][1,4]benzothiazinium chloride (**3**) in a yield of 67% (see Scheme 1). The reaction was performed in a pyridine solution using a 2.5-fold molar excess of aniline.

The reaction proceeds through the nucleophilic attack of the amine on position 4 of the quinoline system, substituting the thiobutyl group with a 4-(trifluoromethyl)phenylamino group. The nucleophilic attack of another amine on the carbonyl carbon leads to the cleavage of the C–S bond and the formation of an intermediate 1-methyl-4-{[4-(trifluoromethyl)phenyl]amino}quinolinium-3-thiolate (**2**). Subsequently, a cyclization reaction occurs, which involves the replacement of the hydrogen atom in the ring of the (trifluoromethyl)phenyl group with a thiolate sulfur atom, as shown in Scheme 1. It should be emphasized that the one-pot procedure without isolation of intermediate product **2** was applied in order to synthesize the final product, compound **3**.

The structure of compound **3** was determined using ^1^H-NMR, ^13^C-NMR spectroscopy and HR-MS spectrometry, respectively. In the ^1^H-NMR spectrum, specified in DMSO solution, the proton signals overlap, which makes the interpretation and confirmation of the analyzed structure rather a dubious task. Therefore, ^1^H-NMR and ^13^C-NMR spectra were collected in CD_3_OD solution. Fortunately, in the ^1^ H-NMR spectrum determined in the CD_3_OD solution, the signals of all protons are separated; however, the amine hydrogen atom signal, which is typical for the spectra of amines in protic solvents, is missing.

In the ^1^H-NMR spectrum, the H8 proton signal appears as a doublet with a coupling constant of ^4^*J* = 1.2 Hz at δ = 7.35–7.38. The H11 proton signal comprises a doublet with a coupling constant of ^3^*J* = 8.4 Hz at δ = 7.32–7.35, while the H10 proton signal forms a doublet of doublets with coupling constants of ^3^*J* = 8.4 Hz and ^4^*J* = 1.2 Hz at δ = 7.46–7.51. Such multiplicity and coupling constants confirm the location of the trifluoromethyl group (-CF_3_) in position 9 of the quinobenzothiazinium system. The application of 2D HSQC and HMBC spectra enabled us to assign signals of all protons and carbon atoms in ^1^H-NMR and ^13^C-NMR spectra, which definitely confirmed the structure of the synthesized molecule **3**. As a matter of fact, the compliance between the calculated value of the molecular weight of the molecular cation and the value obtained in the HR-MS spectrum (to the fourth decimal place) confirms the elemental composition of the analyzed derivative **3**. Finally, the structure of agent **3** was established by X-ray diffraction analysis.

### 2.2. X-ray Analysis

**Figure 4:**
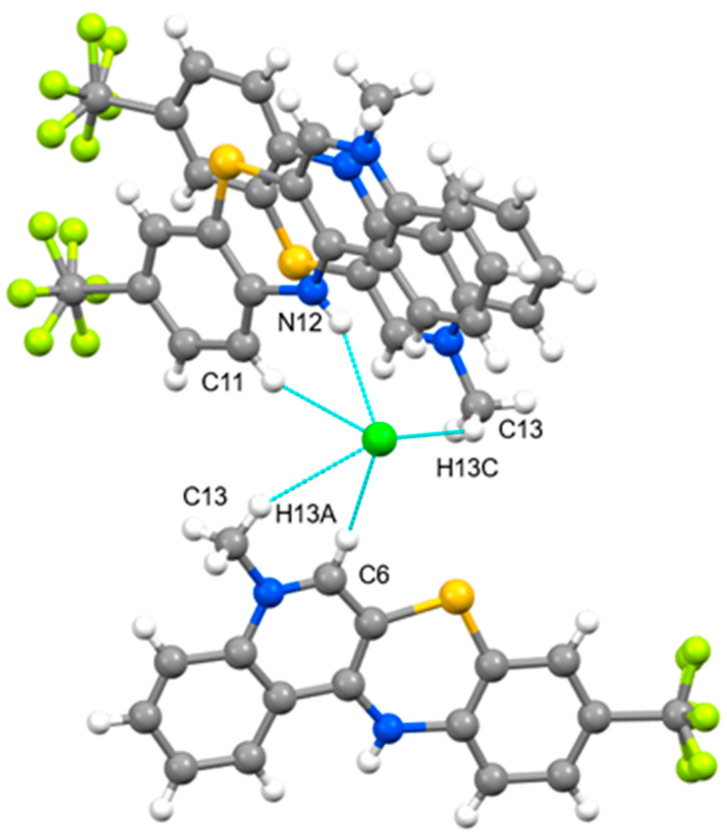
Hydrogen bonds present in the crystal structure.

The single crystal of 5-methyl-9-(trifluoromethyl)-12*H*-quino[3,4-*b*]-[1,4]benzothiazinium chloride (**3**) was formed by crystallization from an ethanol/water mixture in a volume ratio of 1/1. The crystal contains water molecules with s.o.f. = 0.25. The –CF_3_ group is disordered over two positions with s.o.f. equal to 0.6 and 0.4, respectively. The chlorine anion is located close to the N12 nitrogen atom and lies in the plane of the molecule forming one classical N–H···Cl and four weak C–H···Cl hydrogen bonds to three neighboring molecules, as illustrated in Figure 4 and reported in Table 1.

**Figure 5:**
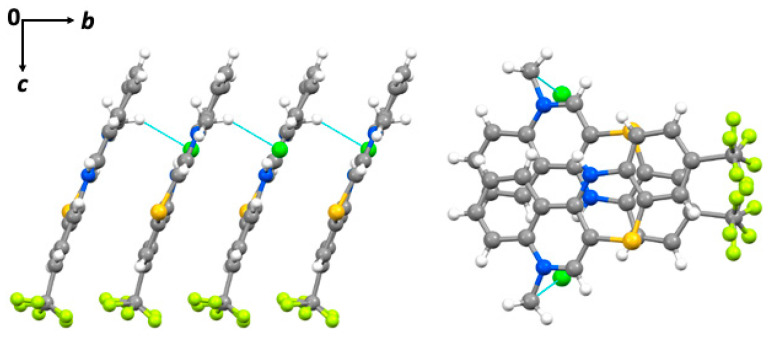
Stacking of molecules in crystal structure: (**left**) view along crystallographic *a* axis showing weak C–H···Cl interactions between molecules; (**right**) view along crystallographic *b* axis.

One of the hydrogen bonds (C13–H13C···Cl1) is formed between parallel-oriented stacks of molecules where stacking occurs along the crystallographic *b* axis. The molecules in the stack are separated by 3.464 Å, suggesting π-π interactions between them, as shown in Figure 5.

**Figure 6:**
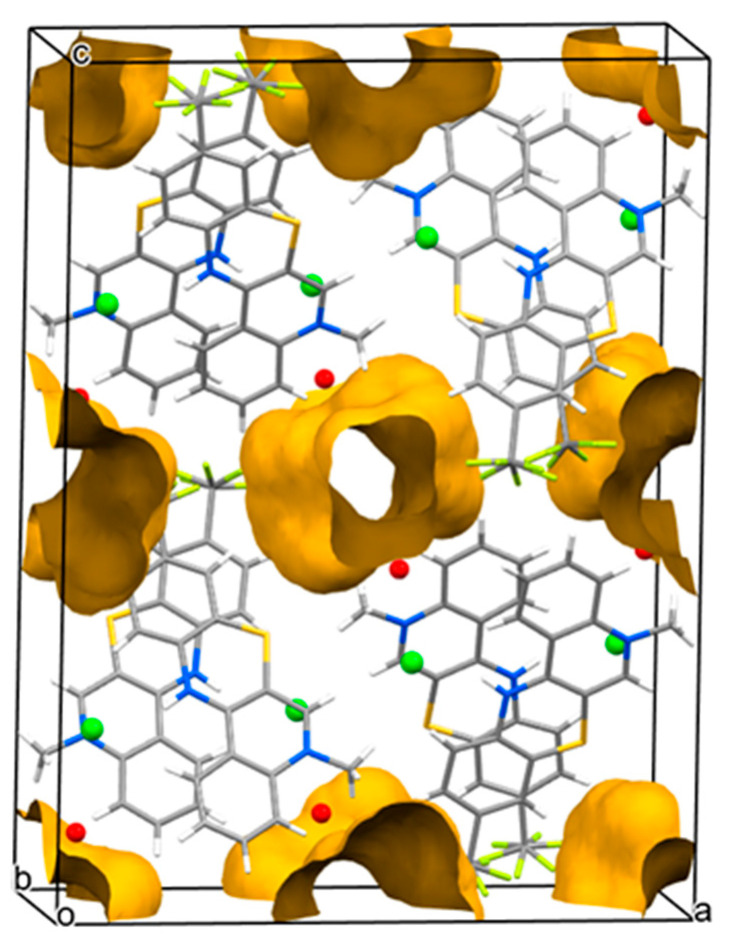
Packing of molecules in unit cell showing channel-type voids running along crystallographic *b* axis.

The packing of the molecules in the unit cell is demonstrated in Figure 6. A detailed examination of the packing mode revealed channel-type voids running along the crystallographic *b* axis in the region where water molecules with low occupancy are present. This suggests that the channels are formed by desorption of solvent molecules from the crystal after drying of the crystals.

Molecule **3** is almost flat; it is only slightly bent along the axis defined by the nitrogen and sulfur atoms of the thiazine ring. The dihedral angle between the planes defined by the nitrogen and sulfur atoms of the benzene ring and the quinoline system is 173.47°. The angle between C6a–S7–C7a atoms in the thiazine ring is 102.3°, and between the C11a–N12–C12a atoms reaches a value of 123.4°. The majority of the geometrical parameters of the molecule are similar to those of the previously described unsubstituted tetracyclic structure of 5-methyl-12*H*-quino[3,4-*b*][1,4]benzothiazinium chloride [27], as reported in Table 2.

### 2.3. DFT Calculations

We performed electronic structure calculations employing the DFT (Density Functional Theory) method to obtain insight into the electronic charge distribution of the studied molecule. We utilized both the crystal structure of molecule **3** and its reoptimized version as presented in the Supplementary Materials. Although both geometries are very similar (an RMSD value of about 0.4 Å), the optimized gas phase structure of **3** is less polar with the value of the calculated dipole moment of 14.29 D, as compared to the value of 18.41 D for the crystal structure. Electronic charge distribution can be conveniently expressed in terms of atomic charges resulting from the population analysis. The results of the Hirshfeld analysis reveal the charge on the Cl atom to be −0.721 for the crystal, and −0.555 for the optimized structure. It also equalized the atomic charges of F (ca. −0.11) for the optimized structure.

In order to study the influence of a solvent on the structure of **3**, we performed calculations using the so-called continuum approach. In this approach, specific solute–solvent interactions were ignored, but overall agreement with the experimental results was expected. In the presented case, the presence of water enhances the polar character of the studied molecule, as can be seen from the value of the dipole moment of 24.09 D and the charge on the Cl atom of −0.736.

### 2.4. In Vitro Antiproliferative Activity

**Figure 7:**
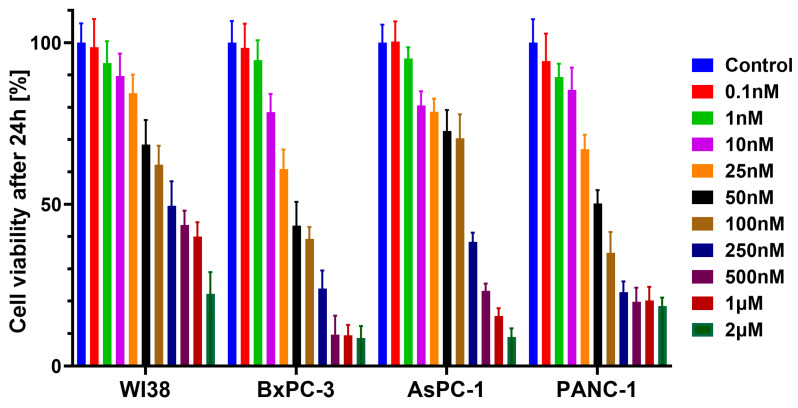
Antiproliferative activity of compound **3** against pancreatic cancer cell lines (Panc-1, AsPC-1 and BxPC-3) and normal fibroblast cell line WI38 in concentration range from 0.1 × 10^−3^ µM to 2 µM with cell viability after 24 h.

**Figure 8:**
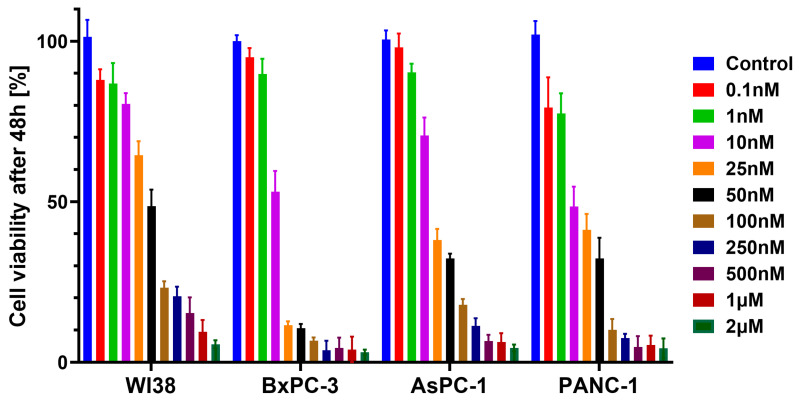
Antiproliferative activity of compound **3** against pancreatic cancer cell lines (Panc-1, AsPC-1 and BxPC-3) and normal fibroblast cell line WI38 in concentration range from 0.1 × 10^−3^ µM to 2 µM with cell viability after 48 h.

A number of tetracyclic quinobenzothiazinium derivatives have been synthesized so far using the presented synthetic methodology [27]. Accordingly, the structure of the compounds was modified by introducing different substituents at different positions of the benzene ring, revealing interesting biological activities. Not surprisingly, the SAR analysis showed that the anticancer activity of the investigated molecules is related to the variations in the molecular structure, e.g., the nature of the introduced substituents and their position in the tetracyclic quinobenzothiazine system. It has been postulated that the mechanism of in vitro antiproliferative activity of quinobenzothiazines may involve DNA intercalation of cancer cells [29]. The investigated compounds demonstrated the ability to form complexes with the DNA of cancer cells. Such a mechanism of anticancer activity is demonstrated by anthracycline antibiotics commonly used in chemotherapy, such as doxorubicin (DOX). The flat structure of the marketed drug molecule appears to facilitate the intercalation of the DNA helix and the formation of stable drug–DNA complexes. Hence, the synthesis of trifluoromethyl quinobenzothiazinium derivative and its X-ray analysis were performed. It is expected that the presence of a strongly electron-withdrawing trifluoromethyl group (–CF_3_) in the quinobenzothiazine system might affect the distribution of electron density and, in consequence, the geometry of the whole molecule (tetra-planarity of the cyclic quinobenzothiazine system), resulting in the improvement of the anticancer potency. In order to determine the antiproliferative activity of quinobenzothiazine **3**, three pancreatic cancer cell lines (Panc-1, AsPC-1 and BxPC-3) and normal fibroblast cell line WI38 were scrutinized meticulously, where the concentration of compound **3** varied in the range from 0.1 × 10^−3^ µM to 2 µM. The detailed findings are presented graphically in Figure 7 with cell viability after 24 h and in Figure 8 with cell after 48 h, respectively.

Compound **3** demonstrated relatively strong antiproliferative potency against all the tested pancreatic cancer cell lines. Extending the exposure time of agent **3** to the tested cell lines resulted in a stronger antiproliferative effect. After only 24 h of exposure, the IC_50_ values varied in the range of 0.046–0.226 µM, as reported in Table 3. It is important to note that the IC_50_ values (0.051 µM and 0.066 µM) against BxPC-3 and Panc-1 carcinoma cells were significantly lower than the effect of compound **3** on cell viability of the non-cancer cell line (IC_50_ = 0.36 µM). The comparison/evaluation statistics of viability of cell line WI38 with respect to pancreatic cancer cell lines and time are shown in Table 4.

It should be emphasized that molecule **3** showed higher anticancer potency compared to DOX used as a reference compound. Interestingly, even greater IC_50_ antiproliferative activity of compound **3** was observed in comparison with the commercially available drug gemcitabine (GEM), a drug commonly used in the treatment of pancreatic cancer. In practice, GEM is the first-line drug in patients with locally advanced (unresectable stage II or III) or metastatic (stage IV) pancreatic adenocarcinoma. Unfortunately, the dense stroma that surrounds pancreatic cancer cells is a characteristic feature of this adenocarcinoma, which makes it extremely difficult to deliver anticancer drugs to cancer cells [32,33,34,35,36,37,38,39,40,41,42,43]. Most of the stroma is formed by the extracellular matrix, which is responsible for its stiffness and increased interstitial tissue pressure. This structure allows cancer cells to survive while simultaneously being a resource of nutrients. Moreover, the stroma also plays a valid role in cancer progression and metastasis [44]. Hence, the issues associated with drug distribution within the tumor environment in pancreatic cancer inspired us to investigate the antiproliferative potency against the selected cell lines. Evidently, the reported anticancer activities should be treated as a starting point for further research of the presented molecules because the details of molecular interactions at the cellular level must be specified in order to determine the mechanism of their actions. Due to its structural similarity to doxorubicin (e.g., flat ring system), compound **3** could be potentially applied in the pharmacological therapies of cancers where the marketed drug (DOX) is successfully employed. Roughly speaking, the presence of fluorine atoms in molecule **3** is a structural pattern shared with another drug (GEM) that can exert a similar damaging impact on the cancer cells. In consequence, further research of compound **3** is planned in order to determine the mechanism of action on a broader spectrum of cancer cell lines. Hopefully, the conducted in vivo studies will confirm the expected effectiveness of the presented molecule **3** in pancreatic cancer therapy.

### 2.5. ANOVA Statistical Analysis

All experiments were repeated three times; therefore, all data are presented as mean ± standard deviation (SD). One-way ANOVA (analysis of variance) implemented in the GraphPad software Prism version 10.0.0 for Windows (Boston, Massachusetts USA, www.graphpad.com) with Šídák’s multiple comparisons test was applied to evaluate the variance between groups. In practice, * *p* ≤ 0.05, ** *p* ≤ 0.01, *** *p* ≤ 0.001 and **** *p* ≤ 0.0001 were considered as statistically important.

As shown in Table 4, regardless of the incubation time and concentration, the statistically significant variations in survival were reported for BxPC-3 in comparison to the WI38 cell line (used as a reference cell line) in the investigated concentration ranges and time, respectively. In the case of the Panc-1 cell line, statistically valid differences were demonstrated in cell proliferation in the concentration range from 25 nM to 1 μM after 24 h of incubation compared to the survival of WI38 cells at the corresponding concentrations and time. Moreover, a significant effect of a longer incubation time (48 h) was observed on the reduction in the number of Panc-1 cells, reaching a statistically significant difference (*p* < 0.0001) at a concentration of 10 nM. As a matter of fact, statistically significant differences were recorded for the AsPC-1 cell line in the concentration range from 100 nM to 1 μM after 24 h of incubation using an analogous reference cell line. Similarly to Panc-1, a significant effect of longer incubation time on the reduction in the number of AsPC-1 cells was observed, reaching a statistically significant difference (*p* < 0.0001) at a concentration of 10 nM.

## 3. Materials and Methods

### 3.1. Chemisty

The melting points were uncorrected. NMR spectra were recorded using a Bruker Ascend 600 spectrometer (Bruker, Billerica, MA, USA). To assign the structures, the following 2D experiments were employed: ^1^H-^13^C gradient selected HSQC and HMBC sequences. Standard experimental conditions and standard Bruker programs were used. The ^1^H-NMR and ^13^C-NMR spectral data were provided relative to the TMS signal at 0.0 ppm. HR mass spectra were recorded with Bruker Impact II (Bruker, Billerica, MA, USA).

### 3.2. Synthesis

#### Synthesis of 5-methyl-9-(trifluoromethyl)-12H-quino[3,4-*b*][1,4]benzothiazinium chloride (**3**)

First, 2.5 mmol (403 mg) 4-(trifluoromethyl)aniline was added to a suspension of 1 mmol (404 mg) 1-methyl-4-butylthio-3-(benzoylthio)quinolinium (**1**) chloride in 5 mL anhydrous pyridine. The resulting mixture was heated at 80 °C for 24 h after being intensively stirred. Then, the mixture was cooled to reach room temperature. The produced precipitate was filtered off under reduced pressure and washed with anhydrous ether (3 × 15 mL). The crude product was purified by recrystallization from anhydrous ethanol.

*5-Methyl-9-(trifluoromethyl)-12H-quino[3,4-b][1,4]benzothiazinium chloride* (**3**): Yield: 67%; ^1^H-NMR (CD_3_OD, 600 MHz), δ (ppm): 4.26 (s, 3H, CH_3_), 7.32–7.35 (d, ^3^*J* = 8.4 Hz, 1H, H11), 7.35–7.38 (d, ^4^*J* = 1.2 Hz, 1H, H8), 7.46-7.51 (dd, ^3^*J* = 8.4 Hz, ^4^*J* = 1.2 Hz, 1H, H10), 7.87–7.92 (m, 1H, H2), 8.08–8.13 (m, 1H, H3), 8.13-8.18 (m, 1H, H4), 8.53 (s, 1H, H6), 8.55–8.59 (m, 1H, H1); ^13^C-NMR (CD_3_OD_,_ 150.9 MHz), δ (ppm): 42.42 (CH_3_), 107.36 (C7a), 116.02 (C12b), 117.97 (C6a), 118.36 (C4), 118.88 (C11), 122.68 (C1), 123.57 (q, ^3^*J*_CF_ = 12 Hz, C8), 124.38 (C9 or CF_3_), 125.54 (q, ^3^*J*_CF_ = 12 Hz, C10), 128.44 (C2), 128.73 (q, *J*_CF_ = 132 Hz, (C9 or CF3), 134.78 (C3), 139.26(C4a), 139.79 (11a), 143.22 (C6), 152.12 (C12a); ESI-HRMS Calcd for C_17_H_12_F_3_N_2_S, (M^+^): 333.0673, found: 333.0673. ^1^H-NMR and ^13^C-NMR spectrum data are reported in Supplementary Materials (see Figures S1 and S2).

### 3.3. X-ray Structural Analysis

C_17_H_12_ClF_3_N_2_S·0.25H_2_O, *M*_r_ = 373.30, red needle, 0.028 × 0.055 × 0.422 mm, orthorhombic, space group *Pbca*, *a* = 19.739(3), *b* = 7.589(1), *c* = 22.555(4) Å, *V* = 3978(1) Å^3^, *Z* = 8, *D*_c_ = 1.265 g/cm^3^, *F*000 = 1576, Bruker AXS D8 VENTURE (Bruker), Mo*Ka* radiation, *λ* = 0.71073 Å, *T* = 296(2) K, 2*θ*_max_ = 56.96°, 135888 reflections collected, 4443 unique (*R*_int_ = 0.098). The structure was solved and refined using the XT, VERSION 2018/2 [45], and SHELXL-2019/1 [46] programs, respectively. Final *GooF* = 0.79, *R* = 0.187, *wR* = 0.356, *R* indices based on 668 reflections with *I* > 2*σ*(*I*) (refinement on *F*^2^), 126 parameters, 0 restraints. Lp and absorption corrections applied, *μ* = 0.325 mm^−1^.

### 3.4. Computational Details

All calculations were carried out using ORCA, an ab initio, DFT and semiempirical SCF-MO package (version 5.0.3) developed by Neese and his group [47]. ORCA was installed and run on a local Linux workstation. Recently, the Density Functional Theory (DFT) has become a work horse for molecular computational chemistry. A review of applications of DFT in spectroscopy is given elsewhere [48,49]. We chose the range-separated hybrid functional theory with dispersion corrections included (ωB97X-D4 [50,51]) and employed the so-called def2-TZVP basis sets of Ahlrichs [52]. The presence of solvent was accounted for by the Gaussian Charge Scheme (C-PCM) implemented in ORCA [53].

### 3.5. Biological Evaluation

#### 3.5.1. Cell Culture

The antiproliferative activities of the investigated compound were assessed using the following three cultured cell lines: Panc-1 (human epithelioid pancreatic carcinoma), AsPC-1 (human pancreatic adenocarcinoma) and BxPC-3 (human primary pancreatic adenocarcinoma), respectively, in addition to normal fibroblast cell line WI38 (obtained from Cell Bank of the Maria Sklodowska-Curie National Research Institute of Oncology in Gliwice). The human pancreatic cancer cell lines Panc-1, AsPC-1 and BxPC-3 were purchased from MERCK (Darmstadt, Germany). Panc–1 was maintained in DMEM containing 10% fetal bovine serum (FBS) (Biowest, Nuaille, France) and 1% of penicillin/streptomycin (Gibco–Thermo Fisher Scientific, Carlsbad, CA, USA) and AsPC–1 and BxPC-3 in RPMI–1640 (Biowest) with 10% FBS with the addition 2% of GlutaMax (Gibco) and 1% of penicillin–streptomycin (Gibco). Cultures grown in a humid atmosphere (temp. 37 °C and 5% CO_2_) were routinely tested for mycoplasma contamination. The medium was changed every 2–3 days. Cell passage was performed when the confluence of cells reached 70–80%.

#### 3.5.2. Proliferation Assay

The antiproliferative effect of the compounds was exerted on cancer and normal cells of the Panc-1, AsPC-1 and BxPC-3 lines, which were seeded in 96-well plates in appropriate media (as described above) in the amount of 4 × 10^3^ cells/well. After 24 h of incubation, the cells were treated with molecule **3** in the concentration range from 0.1 × 10^−3^ µM to 2 µM. In the control cells, the medium was replaced with a new one. The negative control was the medium itself. The cells were incubated for 24 and 48 h at the temperature of 37 °C and 5% CO_2_. After the appropriate incubation time, MTS reagent (Promega, Madison, WI, USA) was added to the wells and incubated for the next 1.5 h.

The MTS test is a colorimetric method for detecting live, proliferating cells. The colored substance is yellow 3-(4,5-dimethylthioazole-2-yl)-5-(3-carboxymethoxyphenyl)-2-(4-sulfophenyl)-2*H*-tetrazole, also known as tetrazolium blue salt, which is transformed by living cells into red-purple formazan. This transformation occurs in metabolically active cells with the participation of the enzyme mitochondrial dehydrogenase, while the amount of formazan produced is proportional to the number of living cells. In this case, the absorbance readings at a wavelength characteristic of formazan allow the determination of the degree of cell survival and, consequently, the calculation of the IC_50_ value (the concentration causing a 50% inhibition of the growth of the cell population). The absorbance was then measured spectrophotometrically at a wavelength of 490 nm using a Tecan microplate reader (Tecan., Mannedorf, Switzerland). Then, the percentage of live cells was calculated according to the following formula:

(1) $$\frac{Absorbance\;of\;samples\;\left[nm\right]-Absorbance\;of\;blank[nm]}{Absorbance\;of\;control\;\left[nm\right]-Absorbance\;of\;blank[nm]}\times 100\%$$

The obtained findings are reported as the means of at least two independent experiments carried out in triplicate. The antiproliferative activity of investigated compound **3** was compared with the following commercially available drugs: gemcitabine and doxorubicin. The values of IC_50_ were specified from the dose–response relationship concerning the control.

## 4. Conclusions

In summary, the reaction of 1-methyl-4-butylthio-3-(benzoylthio)quinolinium chloride (**1**) with 4-(trifluoromethyl)aniline enabled us to synthesize 5-methyl-9-(trifluoromethyl)-12*H*-quino[3,4-*b*][1,4]benzothiazinium chloride (**3**) with the satisfactory yield of 67%. The structure of the product was specified using ^1^H-NMR spectroscopy, ^13^C-NMR spectroscopy, and HR-MS spectrometry. The application of 2D-NMR methodologies (HSQC and HMBC) allows the assignment of signals to all protons and carbon atoms, which confirms the structure of compound **3** in solution. Moreover, the structure of the molecule and the arrangement of molecules in the solid state were determined by X-ray diffraction studies. X-ray analysis showed that the tetracyclic quinobenzothiazinium system is almost flat, with a dihedral angle between the planes formed by the benzene ring and the quinoline system equal to 173.47°. The quantum chemical DFT calculations revealed the polar character of the studied system **3** with the large value of the dipole moment and its significant increase with the presence of water as a solvent. The optimized structures both in gas phase and in solvent will be used in further studies on physicochemical properties of the molecule. Furthermore, the antiproliferative activity of synthesized molecule **3** was tested against the following pancreatic cancer cell lines: Panc-1, AsPC-3, and BxPC-3, respectively. After just 24 h of exposure, the IC_50_ coefficient varied in the range of 0.051–0.222 µM, although it is worth noting that the IC_50_ values against BxPC-3 and Panc-1 cancer cells (0.051 µM and 0.066 µM) were significantly lower than those recorded for the effect of the investigated compound on cell viability of the non-cancer cell line WI38 (IC_50_ = 0.36 µM).

It should be underlined that compound **3** revealed higher anticancer potency compared to doxorubicin (DOX) and gemcitabine (GEM), which were used as reference molecules. This shows that the geometry of the molecule may be decisive for the anticancer activity of (aza)phenothiazine derivatives. The obtained findings are a starting point for further research of presented agent **3** in order to design the potential anticancer drugs in the group of quinobenzothiazine derivatives.

## Figures and Tables

**Table 1:** Hydrogen bond distances (Å) and angles (°) for compound **3**.

	H-Donor	H-Acceptor	Donor-Acceptor	Angle
N12–H12···Cl1	0.86	2.42	3.251(9)	161.6
C6–H6···Cl1 ^1^	0.93	2.64	3.53(1)	160.1
C11–H11···Cl1	0.93	2.67	3.48(1)	146.8
C13–H13A···Cl1 ^1^	0.96	2.88	3.77(1)	154.7
C13–H13C···Cl1 ^2^	0.96	2.70	3.47(1)	137.4

Symmetry equivalent positions: ^1^ *x* − 1/2, *y*, −*z* + 3/2; ^2^ −*x* + 1/2, *y* + 1/2, *z*.

**Table 2:** Selected bond lengths [Å] and angles [deg.] for molecule **3**.

Bond Lengths [Å]			
S7–C7A	1.704(11)	N5–C6	1.275(12)
S7–C6A	1.763(12)	N5–C4A	1.385(10)
C12A–C6A	1.455(14)	N12–C12A	1.382(12)
N12–C11A	1.465(12)	C12A–C12B	1.437(13)
C11A–C7A	1.367(12)	C4A–C12B	1.340(11)
C6–C6A	1.386(12)	C11–C11A	1.441(13)
C9–C14	1.630(18)	N5–C13	1.416(12)
**Bond Angles [°]**			
C7A–S7–C6A	102.3(6)	N5–C6–C6A	119.5(11)
C7A–C11A–N12	121.5(11)	C6–N5–C4A	124.7(10)
N12–C12A–C12B	121.8(10)	C12A–C6A–S7	122.2(9)
C12B–C12A–C6A	115.4(10)	C12B–C4A–N5	119.5(10)
C12A–N12–C11A	123.4(10)	N12–C12A–C6A	122.4(11)
C4A–C12B–C12A	120.4(10)	C6–C6A–S7	117.3(9)
C6–C6A–C12A	120.2(11)	C11A–C7A–S7	126.9(10)

**Table 3:** In vitro antiproliferative activity (IC_50_ [µM] ± SD, n = 6) against human pancreatic cancer cell lines and cell viability of normal human lung fibroblasts of compound **3** compared to doxorubicin (DOX) and gemcitabine (GEM).

Comp.	IC_50_ [µM]			
	Panc-1	AsPC-1	BxPC-3	WI38
3	0.066 ± 0.03	0.222 ± 0.05	0.051 ± 0.07	0.36 ± 0.1
DOX	0.16 ± 0.02	1.829 ± 0.001	0.159 ± 0.005	-
GEM	0.022 ± 0.01	3.772 ± 0.03	0.025 ± 0.01	0.01 ± 0.01

Panc-1 = human epithelioid pancreatic carcinoma; AsPC-1 = human pancreatic adenocarcinoma; BxPC-3 = human primary pancreatic adenocarcinoma; WI38 = non-cancer human lung fibroblasts.

**Table 4:** Statistical significance of cell viability between normal fibroblasts WI38 and pancreatic cancer cells (PCCs) during 24 and 48 h assays.

Cell Line	Time [h]	P WI38 vs. PCCs at Selected Concentrations						
		10 nM	25 nM	50 nM	100 nM	250 nM	500 nM	1 μM
Panc-1	24	0.7375	<0.0001	<0.0001	<0.0001	<0.0001	<0.0001	<0.0001
	48	<0.0001	<0.0001	<0.0001	<0.0001	<0.0001	<0.0001	0.4932
AsPC-1	24	0.0084	0.3019	0.7226	0.0264	0.0005	<0.0001	<0.0001
	48	<0.0001	<0.0001	<0.0001	0.0158	<0.0001	<0.0001	0.4502
BxPC-3	24	0.0018	<0.0001	<0.0001	<0.0001	<0.0001	<0.0001	<0.0001
	48	<0.0001	<0.0001	<0.0001	<0.0001	<0.0001	<0.0001	0.0199

P—statistical significance of compared cell viability.

## Data Availability

Data are contained within the article. Crystal data for structure **3** are available at the Cambridge Crytallographic Data Centre under CCDC deposition number 2333369 (https://ccdc.cam.ac.uk/).

## Supplementary Materials

The following supporting information can be downloaded at: https://www.mdpi.com/article/10.3390/molecules29184337/s1, Figure S1. ^1^H NMR spectrum of 5-methyl-9-(trifluoromethyl)-12*H*-quino[3,4-*b*][1,4]benzothiazinium chloride (**3**) in CD_3_OD. Figure S2. ^13^C NMR spectrum of 5-methyl-9-(trifluoromethyl)-12*H*-quino[3,4-*b*][1,4]benzothiazinium chloride (**3**) in CD_3_OD. Figure S3. HSQC spectrum of 5-methyl-9-(trifluoromethyl)-12*H*-quino[3,4-*b*][1,4]benzothiazinium chloride (**3**) in CD_3_OD. Figure S4. HMBC spectrum of 5-methyl-9-(trifluoromethyl)-12*H*-quino[3,4-*b*][1,4]benzothiazinium chloride (**3**) in CD_3_OD. Figure S5. ^19^F-NMR spectrum of 5-methyl-9-(trifluoromethyl)-12*H*-quino[3,4-*b*][1,4]benzothiazinium chloride (**3**) in CD_3_OD. Figure S6. HOMO (**a**) and LUMO (**b**) orbitals for molecule **3** optimized in the gas phase. Table S1. HOMO-LUMO gap, electric dipole momentum. HOMO-LUMO gap in eV, dipole moment in Debyes.
