# Supplementary material for: Synthesis and Structure of 5-Methyl-9-(trifluoromethyl)-12H-quino[3,4-b][1,4]benzothiazinium Chloride as Anticancer Agent
Source: Molecules. 2024 Sep 12;29(18):4337. doi: 10.3390/molecules29184337 (PMC11433864; doi:10.3390/molecules29184337)
Supplement: Supplementary file 1 [file molecules-29-04337-s001.zip › molecules-3213270-supplementary.pdf]

Supplementary Materials

Synthesis and Structure of 5-Methyl-9-(trifluoromethyl)-12*H*-quino[3,4-*b*][1,4]benzothiazinium Chloride as Anticancer Agent

Andrzej Zieba [1,*], Violetta Kozik [2,*], Kinga Suwinska [3], Agata Kawulok [2,4], Tadeusz Pluta [2], Josef Jampilek [2] and Andrzej Bak [2]

[1] Department of Organic Chemistry, Faculty of Pharmaceutical Sciences in Sosnowiec, Medical University of Silesia, Jagiellonska 4, 41-200 Sosnowiec, Poland
[2] Institute of Chemistry, University of Silesia, Szkolna 9, 40-007 Katowice, Poland; agata.hadrys@us.edu.pl (A.K.); tadeusz.pluta@gmail.com (T.P.); josef.jampilek@gmail.com (J.J.); andrzej.bak@us.edu.pl (A.B.)
[3] Faculty of Mathematics and Natural Sciences, Cardinal Stefan Wyszynski University, K. Woycickiego 1/3, 01-938 Warszawa, Poland; k.suwinska@uksw.edu.pl
[4] Department of Bone Marrow Transplantation and Oncohematology, Maria Sklodowska-Curie National Research Institute of Oncology, Gliwice Branch, ul. Wybrzeze Armii Krajowej 15, 44-101 Gliwice, Poland
* Correspondence: zieba@sum.edu.pl (A.Z.); violetta.kozik@us.edu.pl (V.K.)

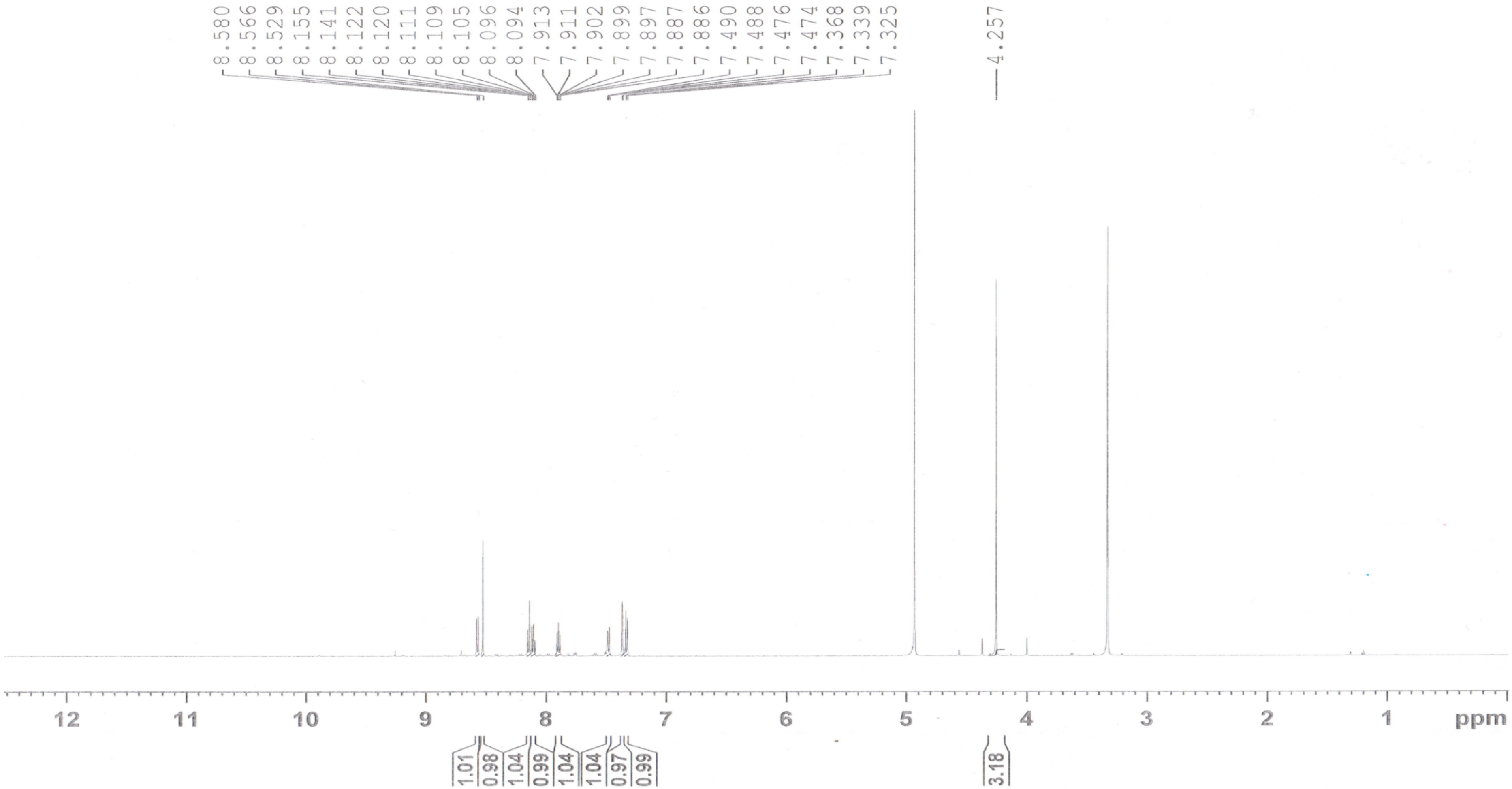

Figure S1. ^{1}H NMR spectrum of 5-methyl-9-(trifluoromethyl)-12*H*-quino[3,4-*b*][1,4]benzothiazinium chloride (3) in CD_3OD.

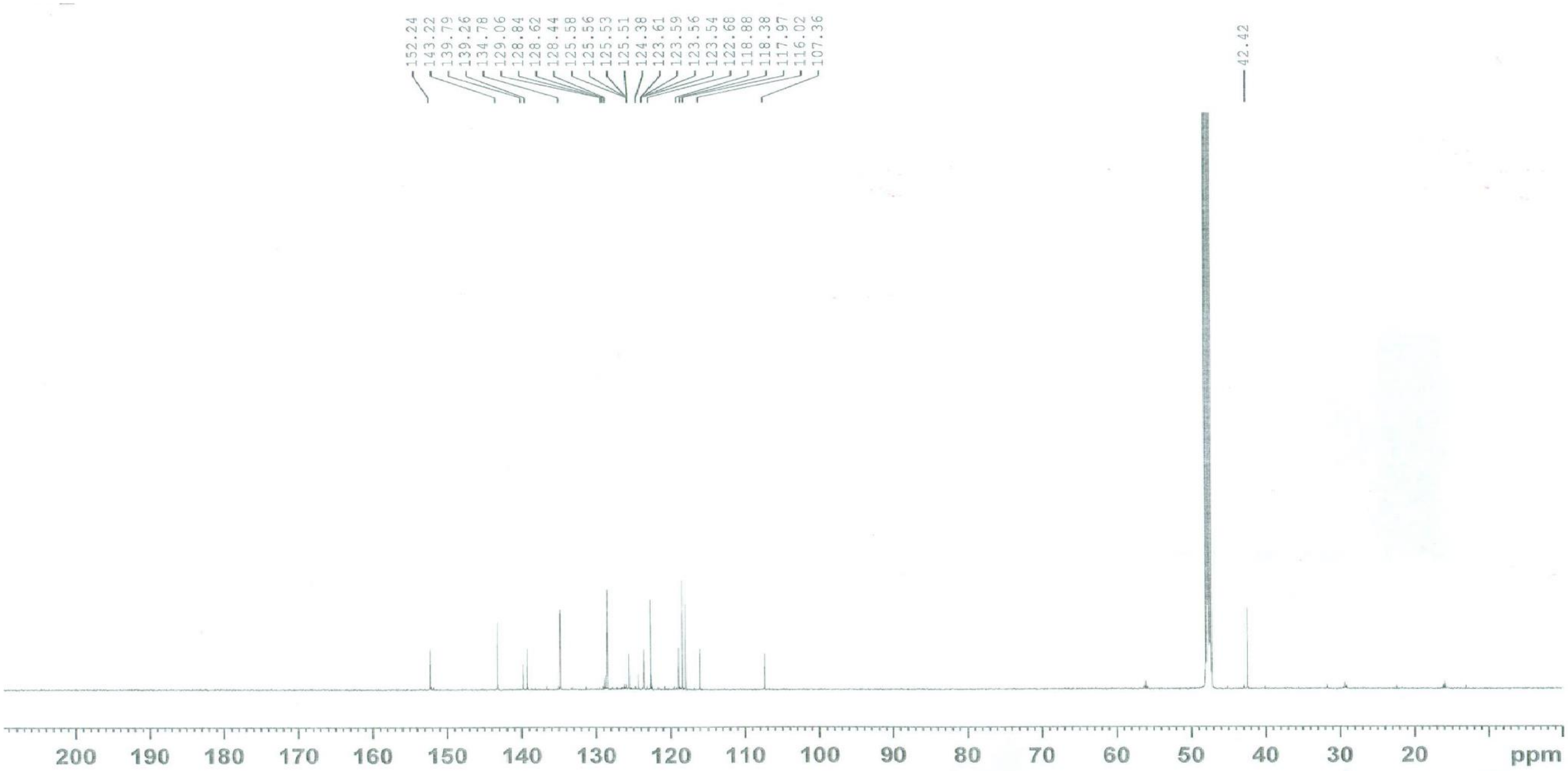

Figure S2. ^{13}C NMR spectrum of 5-methyl-9-(trifluoromethyl)-12*H*-quino[3,4-*b*][1,4]benzothiazinium chloride (3) in CD_3OD.

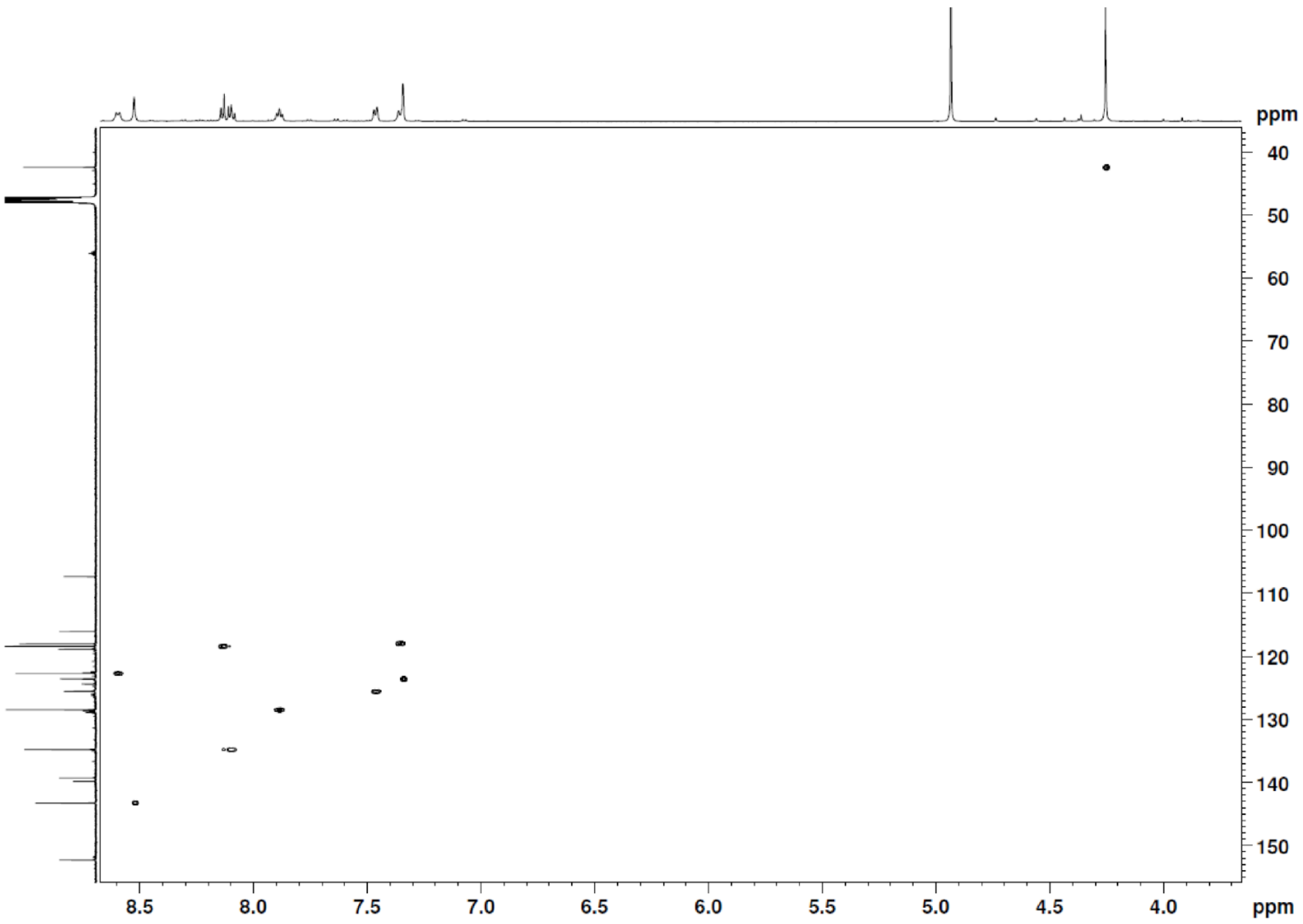

Figure S3. HSQC spectrum of 5-methyl-9-(trifluoromethyl)-12*H*-quino[3,4-*b*][1,4]benzothiazinium chloride (3) in CD_3OD.

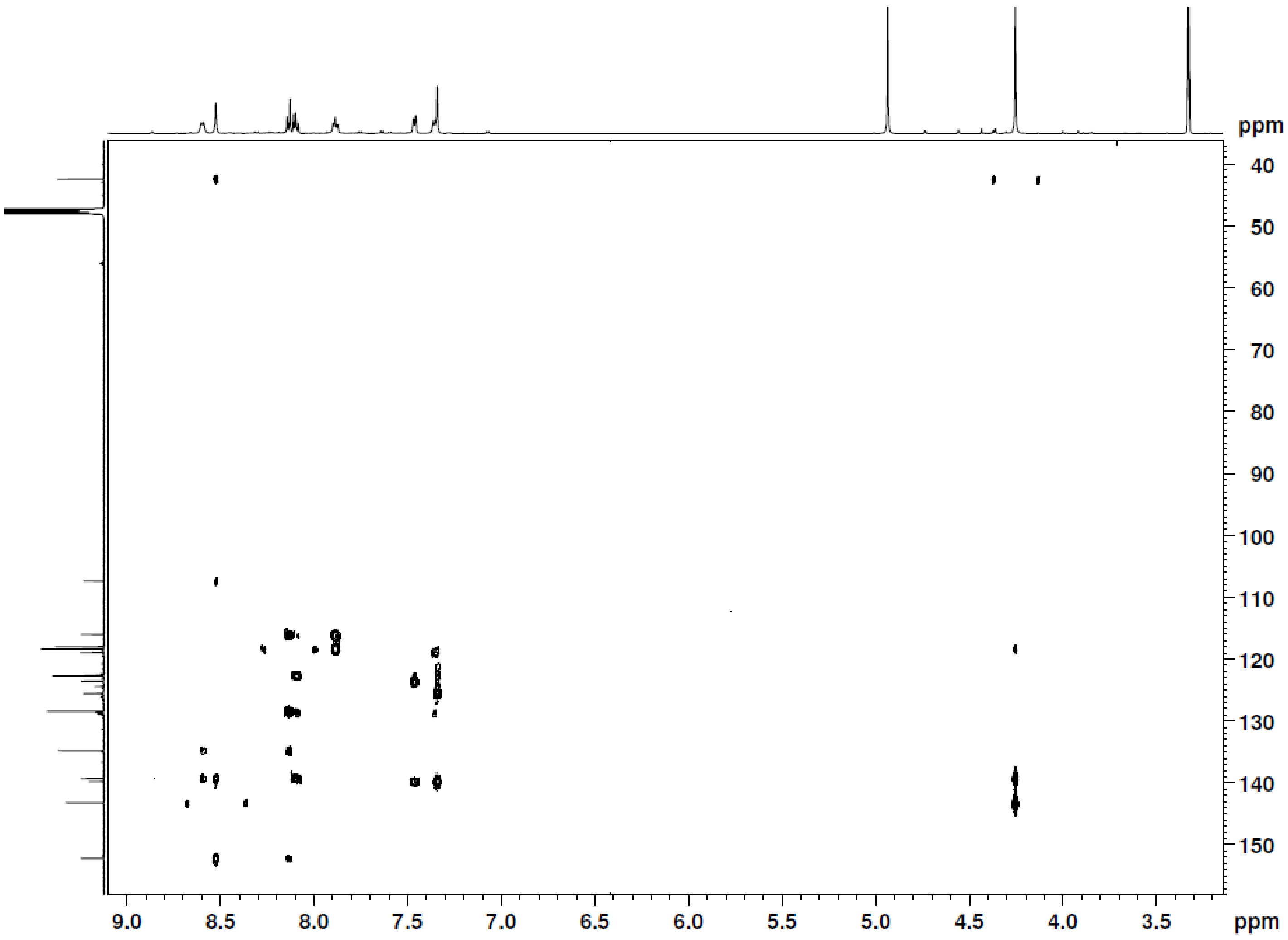

Figure S4. HMBC spectrum of 5-methyl-9-(trifluoromethyl)-12*H*-quino[3,4-*b*][1,4]benzothiazinium chloride (3) in CD_3OD.

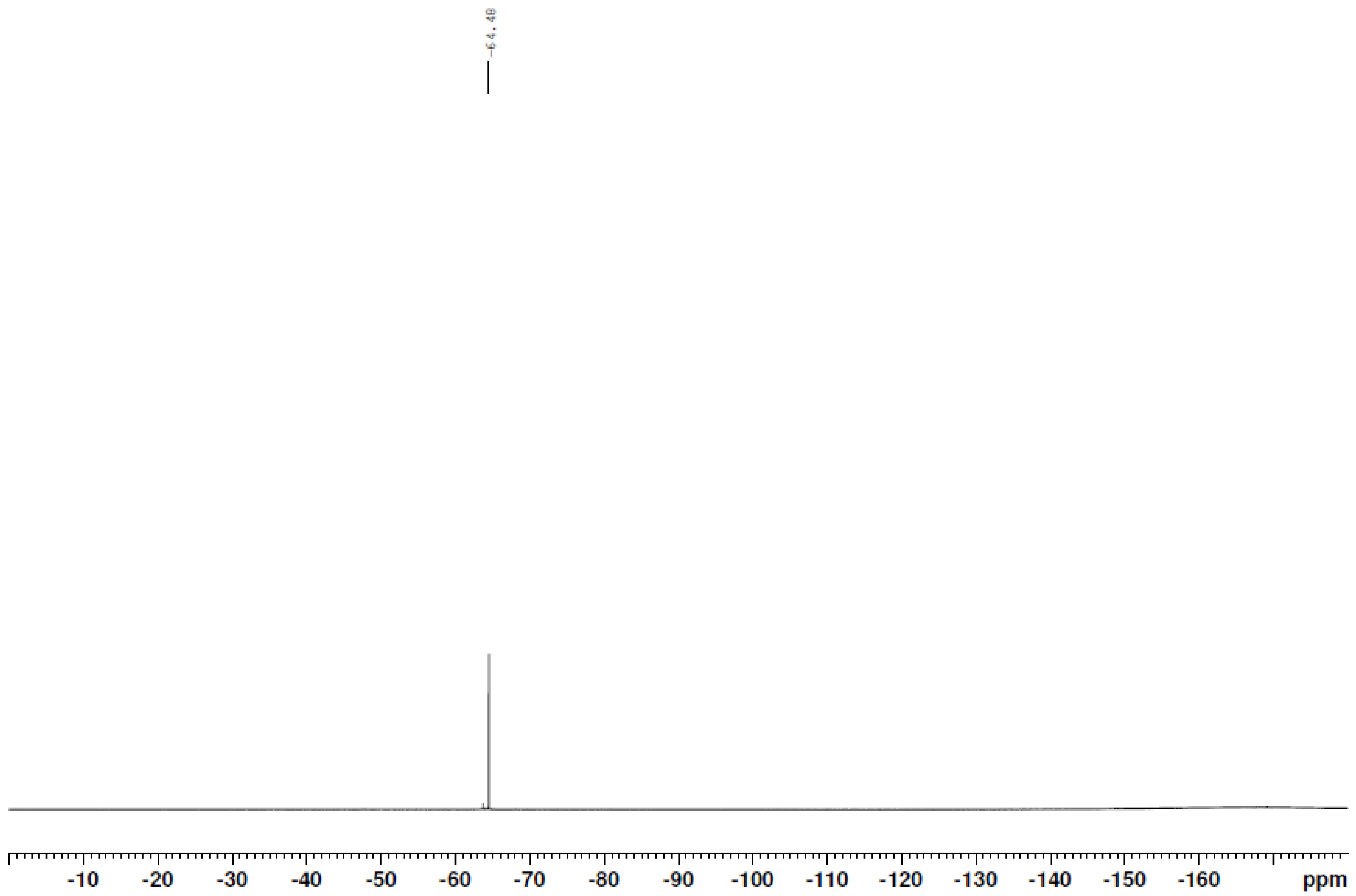

Figure S5. ^{19}F-NMR spectrum of 5-methyl-9-(trifluoromethyl)-12*H*-quino[3,4-*b*][1,4]benzothiazinium chloride (3) in CD_3OD.

Charge distribution of the studied molecule (**3**) as given by the Hirshfeld population analysis.

a) Crystal structure as given (cif format)

Total integrated alpha density = 93.999921339

Total integrated beta density = 93.999921339

ATOM	CHARGE	SPIN
0 Cl	-0.721181	0.000000
1 S	-0.000790	0.000000
2 F	-0.178764	0.000000
3 N	0.015460	0.000000
4 N	-0.061465	0.000000
5 H	0.085324	0.000000
6 C	-0.040860	0.000000
7 H	0.034464	0.000000
8 C	0.073411	0.000000
9 C	0.063967	0.000000
10 H	0.059796	0.000000
11 C	-0.011102	0.000000
12 C	-0.014286	0.000000
13 H	0.044113	0.000000
14 C	0.009981	0.000000
15 H	0.031806	0.000000
16 C	0.071052	0.000000
17 C	-0.003094	0.000000
18 H	0.057687	0.000000
19 H	0.050632	0.000000
20 H	0.051218	0.000000
21 C	0.133741	0.000000
22 C	-0.000017	0.000000
23 C	0.302284	0.000000
24 C	-0.029918	0.000000
25 C	-0.048109	0.000000
26 H	0.026453	0.000000
27 C	-0.007715	0.000000

28 C	0.018856	0.000000
29 H	0.026259	0.000000
30 C	0.003019	0.000000
31 H	0.051441	0.000000
32 C	0.005245	0.000000
33 H	0.044276	0.000000
34 F	-0.077720	0.000000
35 F	-0.065308	0.000000
TOTAL	0.000157	0.000000

b) Structure optimized in the gas phase

Total integrated alpha density = 94.000008501

Total integrated beta density = 94.000008501

ATOM	CHARGE	SPIN
0 Cl	-0.555604	0.000000
1 S	-0.010525	0.000000
2 F	-0.110031	0.000000
3 N	-0.020858	0.000000
4 N	-0.059394	0.000000
5 H	0.086556	0.000000
6 C	-0.051977	0.000000
7 H	0.039067	0.000000
8 C	0.065958	0.000000
9 C	0.055530	0.000000
10 H	0.061847	0.000000
11 C	-0.028269	0.000000
12 C	-0.018663	0.000000
13 H	0.051079	0.000000
14 C	-0.017120	0.000000
15 H	0.041364	0.000000
16 C	0.063800	0.000000
17 C	0.009934	0.000000
18 H	0.053451	0.000000
19 H	0.048536	0.000000

20 H	0.050244	0.000000
21 C	0.123695	0.000000
22 C	-0.009236	0.000000
23 C	0.289377	0.000000
24 C	-0.038590	0.000000
25 C	-0.034500	0.000000
26 H	0.042175	0.000000
27 C	-0.011469	0.000000
28 C	-0.003510	0.000000
29 H	0.032422	0.000000
30 C	-0.020978	0.000000
31 H	0.054722	0.000000
32 C	-0.006188	0.000000
33 H	0.050456	0.000000
34 F	-0.105794	0.000000
35 F	-0.117525	0.000000
TOTAL	-0.000017	0.000000

c) Structure optimized in the presence of the water molecule. Continuum C-PCM scheme applied.

Total integrated alpha density = 93.999869974

Total integrated beta density = 93.999869974

ATOM	CHARGE	SPIN
0 Cl	-0.736462	0.000000
1 S	-0.012725	0.000000
2 F	-0.120723	0.000000
3 N	0.015309	0.000000
4 N	-0.078043	0.000000
5 H	0.096428	0.000000
6 C	-0.030811	0.000000
7 H	0.063273	0.000000
8 C	0.080234	0.000000
9 C	0.097557	0.000000
10 H	0.092716	0.000000
11 C	-0.036544	0.000000

12	C	-0.022811	0.000000
13	H	0.059310	0.000000
14	C	-0.035055	0.000000
15	H	0.035377	0.000000
16	C	0.065442	0.000000
17	C	0.025042	0.000000
18	H	0.071318	0.000000
19	H	0.064856	0.000000
20	H	0.064446	0.000000
21	C	0.118126	0.000000
22	C	-0.005047	0.000000
23	C	0.290651	0.000000
24	C	-0.030382	0.000000
25	C	-0.027132	0.000000
26	H	0.055296	0.000000
27	C	-0.013198	0.000000
28	C	-0.024257	0.000000
29	H	0.022451	0.000000
30	C	-0.026015	0.000000
31	H	0.058463	0.000000
32	C	-0.004514	0.000000
33	H	0.064210	0.000000
34	F	-0.108235	0.000000
35	F	-0.128292	0.000000
TOTAL		0.000260	0.000000

Table S1. HOMO-LUMO gap, electric dipole momentum. HOMO-LUMO gap in eV, dipole moment in Debyes.

Structure	HOMO-LUMO [eV]	Dipole moment [Debyes]
**a)**	1.291	18.41
**b)**	1.315	14.29
**c)**	1.304	24.09

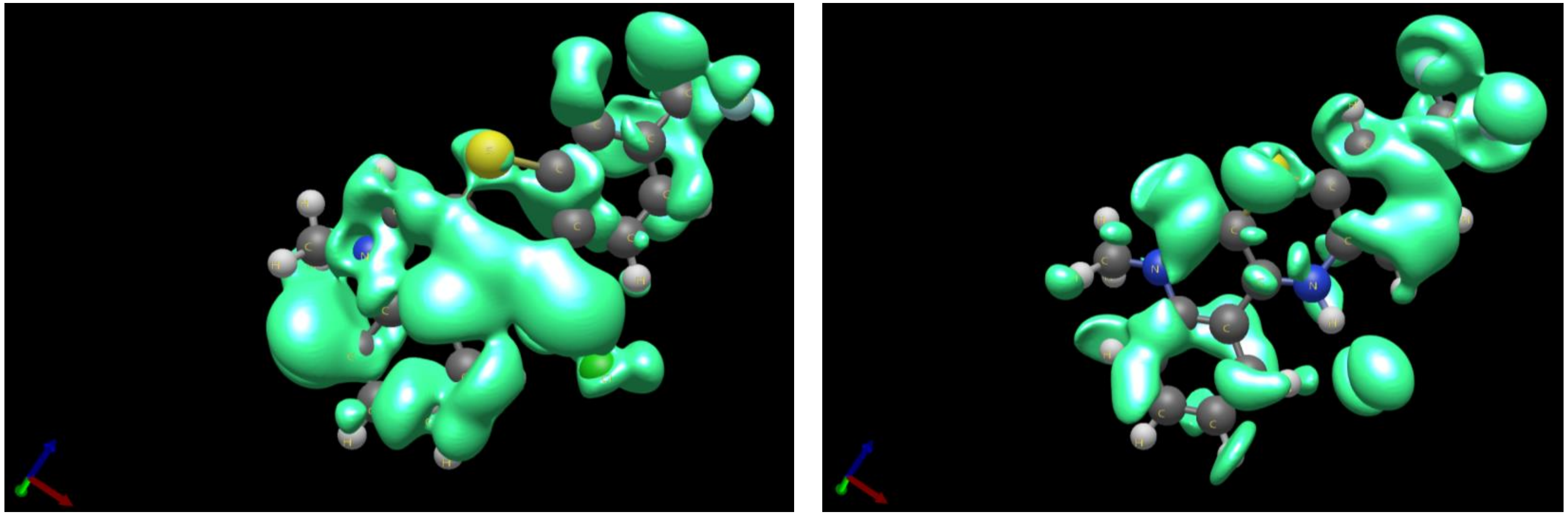

(**a**) (**b**)

Figure S6. HOMO (**a**) and LUMO (**b**) orbitals for molecule **3** optimized in the gas phase.
